# *In Vitro* Evaluation of the Antiviral Activity of the Synthetic Epigallocatechin Gallate Analog-Epigallocatechin Gallate (EGCG) Palmitate against Porcine Reproductive and Respiratory Syndrome Virus

**DOI:** 10.3390/v6020938

**Published:** 2014-02-21

**Authors:** Chunjian Zhao, Shuaihua Liu, Chunying Li, Lei Yang, Yuangang Zu

**Affiliations:** 1 State Engineering Laboratory for Bio-Resource Eco-Utilization, Northeast Forestry University, Harbin 150040, China; E-Mails: zcjsj@163.com (C.Z.); woshiliushuaihua@163.com (S.L.); ylnefu@163.com (L.Y.); 2 Key Laboratory of Forest Plant Ecology, Ministry of Education, Northeast Forestry University, Harbin 150040, China; 3 Engineering Research Center of Forest Bio-Preparation, Ministry of Education, Northeast Forestry University, Harbin 150040, China

**Keywords:** EGCG palmitate, synthesized, anti-PRRSV, MARC-145 cells

## Abstract

In this study, epigallocatechin gallate (EGCG) palmitate was synthesized and its anti-porcine reproductive and respiratory syndrome virus (PRRSV) activity was studied. Specifically, EGCG palmitate was evaluated for its ability to inhibit PRRSV infection in MARC-145 cells when administered as pre-, post-, or co-treatment. EGCG and ribavirin were used as controls. The results showed that a 50% cytotoxic concentration (CC50) of EGCG, EGCG palmitate, and ribavirin was achieved at 2,359.71, 431.42, and 94.06 μM, respectively. All three drugs inhibited PRRSV in a dose-dependent manner regardless of the treatment protocol. EGCG palmitate exhibited higher cytotoxicity than EGCG, but lower cytotoxicity than ribavirin. EGCG palmitate anti-PRRSV activity was significantly higher than that of EGCG and ribavirin, both as pre-treatment and post-treatment. Under the former conditions and a tissue culture infectious dose of 10 and 100, the selectivity index (SI) of EGCG palmitate in the inhibition of PRRSV was 3.8 and 2.9 times higher than that of ribavirin when administered as a pre-treatment, while the SI of EGCG palmitate in the inhibition of PRRSV was 3.0 and 1.9 times higher than ribavirin when administered as a post-treatment. Therefore, EGCG palmitate is potentially effective as an anti-PRRSV agent and thus of interest to the pharmaceutical industry.

## 1. Introduction

Porcine reproductive and respiratory syndrome (PRRS), commonly known as blue-eared pig disease, is an infectious disease characterized by severe reproductive deficiency in pregnant sows and by respiratory symptoms in piglets [1]. It is caused by porcine reproductive and respiratory syndrome virus (PRRSV), an important swine pathogen which, since its discovery in the early 1990s, has quickly spread throughout pig-breeding countries, causing large economic losses [2,3]. PRRSV is an enveloped, single-stranded, positive-sense RNA virus and a member of the order Nidovirales, family Arteriviridae [4]. As a macrophage-tropic virus, it establishes a chronic infection in these cells *in vivo* and replicates in primary pig macrophages *in vitro* [5]. Pigs persistently infected with PRRSV develop viremia, with reduced cellular immunity [6]. The main routes of PRRS infection are respiratory transmission, airborne transmission, airborne spread, contact transmission, and semen transmission. Current antiviral strategies fail to prevent and control PRRSV, such that infected pigs typically become long-term carriers of the virus [7]. Thus, there is a clear need to develop effective anti-PRRSV drugs.

(−)-Epigallocatechin-3-gallate (EGCG), the major catechin extracted from tea, exhibits potent inhibitory effects on many viruses, such as influenza virus, hepatitis B virus, hepatitis C virus (HCV), and human immunodeficiency virus (HIV) [8,9,10,11,12,13,14]. In preliminary experiments, EGCG demonstrated anti-PRRSV activity *in vitro*, although significant effects were achieved only at relatively high concentrations, probably because of the drug’s poor chemical stability and rapid decomposition [15]. Esterification of EGCG is an effective means to improve its stability [16]. Ribavirin is a well-known and broad-spectrum antiviral drug that was previously shown to dose-dependently inhibit PRRSV replication [17,18,19]. Thus, in the present study, the anti-PRRSV activity of EGCG palmitate was evaluated. EGCG and ribavirin served as the positive controls.

## 2. Results

### 2.1. Chemical Structure of EGCG Palmitate

**Figure 1:**
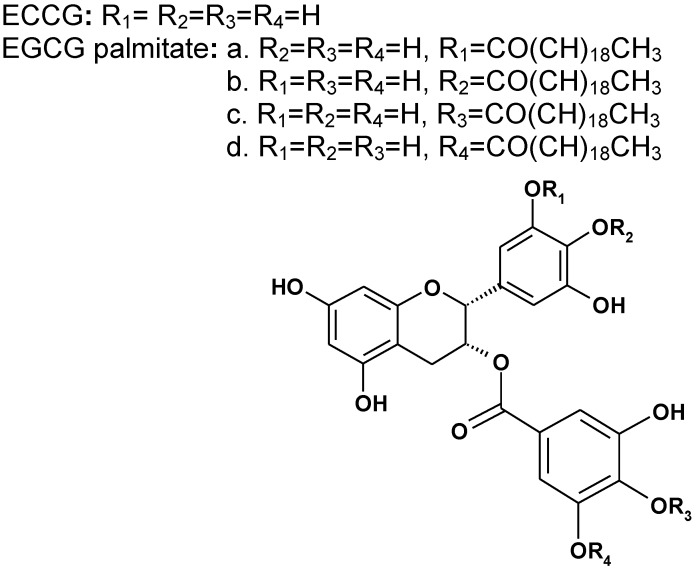
The chemical structure of epigallocatechin gallate (EGCG) and EGCG palmitate.

An EGCG derivative was synthesized and identified as EGCG palmitate by MS, IR, UV, and ^1^H-NMR. EGCG palmitate is composed of a mixture of four regioisomers (a:b:c:d = 20:20:18:44); its chemical structure is shown in Figure 1 and is consistent with that previously reported [20].

### 2.2. Cytotoxicity of EGCG, EGCG Palmitate, and Ribavirin

**Figure 2:**
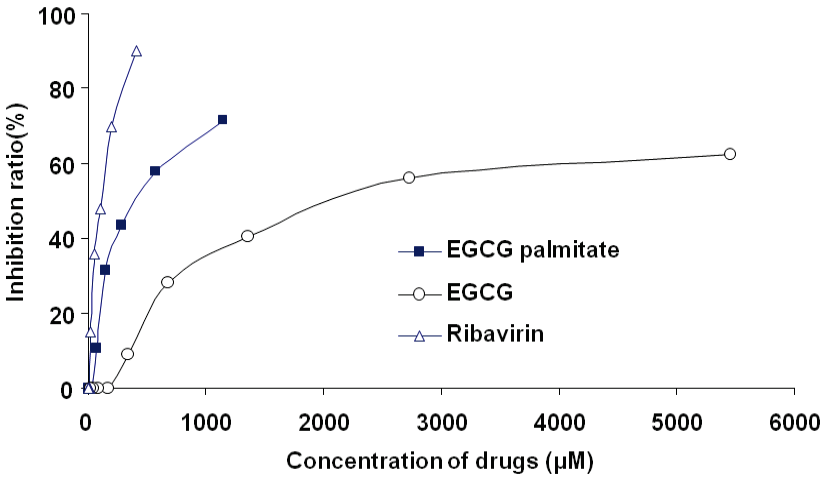
Cytotoxicity of EGCG, EGCG palmitate, and ribavirin in MARC-145 cells.

The cytotoxicity of different concentrations of EGCG (0–5452.75 μM), EGCG palmitate (0–1148.80 μM), and ribavirin (0–409.84 μM) in MARC-145 cells was tested in an MTT assay. The cells were treated with each of the three drugs for 72 h, after which the cell inhibition ratio was evaluated. As shown in Figure 2, the inhibitory effect of EGCG, EGCG palmitate, and ribavirin on cell growth was dose-dependent, with a 50% cytotoxic concentration (CC50) of 2359.71, 431.42, and 94.06 μM, respectively.

**Figure 3:**
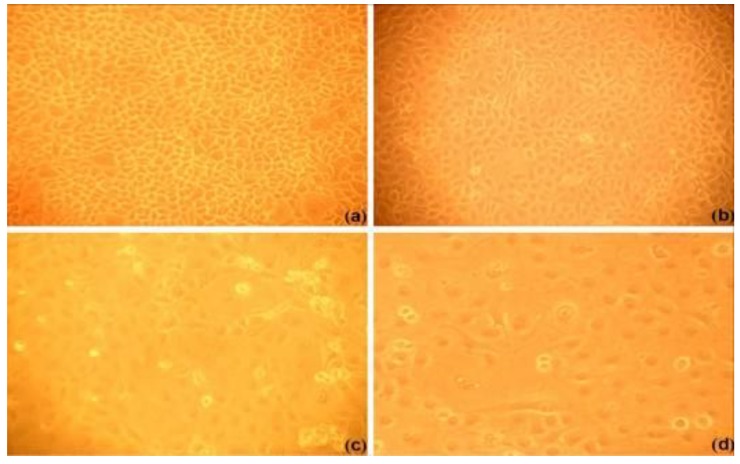
Optical microscopy photographs of cultured MARC-145 cells (×100). The cells were incubated with (**a**) cell culture medium (control group), and either 17.95 μM (**b**), 287.20 μM (**c**), or 1148.80 μM (**d**) EGCG palmitate.

Figure 3 shows the morphology of the treated MARC-145 cells. Compared with the controls (Figure 3a), EGCG palmitate, at a concentration of 17.95 μM (Figure 3b), did not show any proliferation-inhibiting activity. As the concentration of EGCG palmitate increased, the gaps between the plated cells increased as well (Figure 3c,d). Since 17.95 μM EGCG palmitate did not induce obvious cell toxicity; it was used as the maximum initial concentration in further experiments. For EGCG and ribavirin, the corresponding concentrations were 170.40 μM and 12.81 μM, respectively.

### 2.3. The TCID50 of PRRSV

The final cytopathic effects (CPE) resulting from the different concentrations of PRRSV were recorded until the infected cells no longer developed lesions and the control cells were still intact and clearly distinguishable. As shown in Table 1, the 50% tissue culture infective dose (TCID50) according to the Reed–Muench method was 10–3.85 [21].

**Table 1:** Cytopathic effect of porcine reproductive and respiratory syndrome virus (PRRSV) on MARC-145 cells.

Dilution	Infected well	Uninfected well	Cumulative infected well	Cumulative uninfected well	Infected (%)
10^−1^	6	0	20	0	100 (20/20)
10^−2^	6	0	14	0	100 (14/14)
10^−3^	5	1	9	1	90 (9/10)
10^−4^	3	3	3	4	42.8 (3/7)
10^−5^	0	6	0	10	0 (0/10)
10^−6^	0	6	0	16	0 (0/16)
10^−7^	0	6	0	22	0 (0/22)
10^−8^	0	6	0	28	0 (0/28)
10^−9^	0	6	0	28	0 (0/28)

### 2.4. Cytopathic Effect of PRRSV on MARC-145 Cells Pre-Treated with the Test and Control Compounds

**Figure 4:**
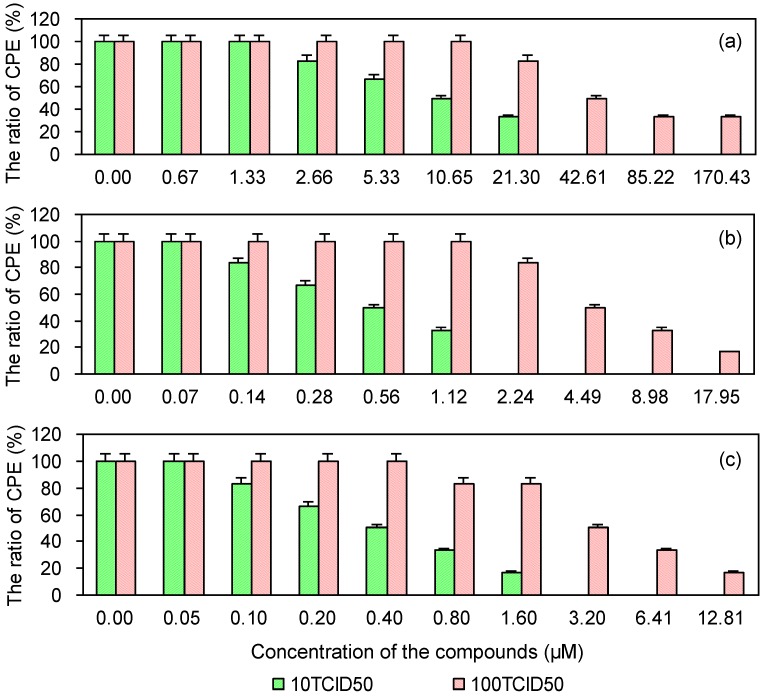
Cytopathic effect of PRRSV on MARC-145 cells pre-treated with EGCG (**a**), EGCG palmitate (**b**), and ribavirin (**c**).

To investigate whether PRRSV infection is reduced in MARC-145 cells pretreated with either EGCG, EGCG palmitate, or ribavirin, a CPE method was used. As shown in Figure 4, the three compounds reduced PRRSV infection in a dose-dependent manner. Their EC50 and selectivity index (SI) values in reducing PRRSV infection are provided in Table 2, which shows that EGCG palmitate more strongly reduced PRRSV infection than either EGCG or ribavirin, in accordance with their SIs.

**Table 2:** Cytopathic effect of PRRSV on MARC-145 cells pre-treated with the test and control compounds.

Compounds	10 TCID_50_		100 TCID_50_	
	EC_50_ (μM) ^a^	SI ^b^	EC_50_ (μM)	SI
EGCG	8.53	276.62	60.25	39.16
EGCG palmitate	0.48	892.29	5.53	77.96
Ribavirin	0.40	234.98	3.48	27.02

^a^ EC_50_ is the concentration that reduced the virus-induced CPE by 50%; ^b^ SI (selectivity index) is the ratio of CC_50_ to EC_50_.

### 2.5. Cytopathic Effect of PRRSV in MARC-145 Cells Post-Treated with the Test and Control Compounds

**Figure 5:**
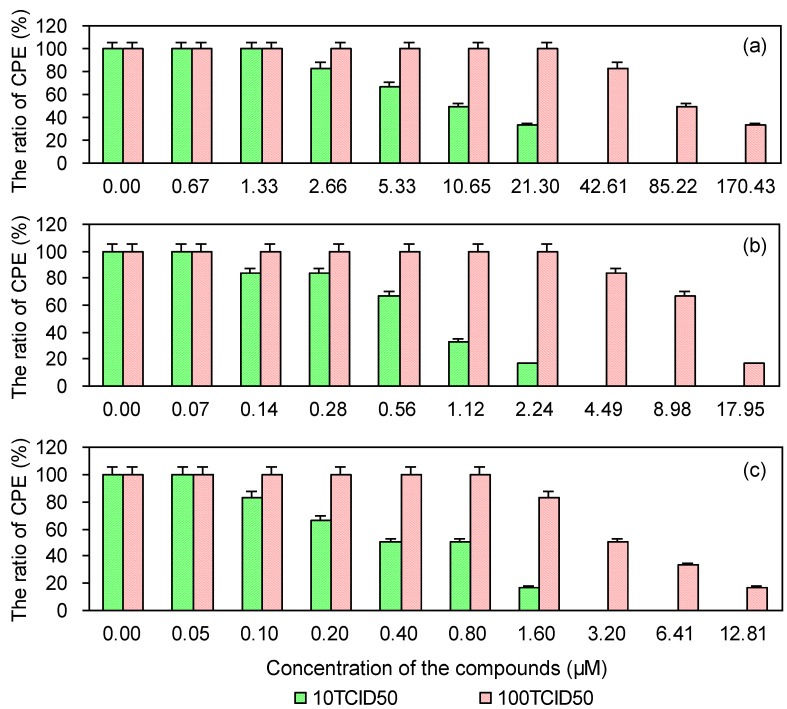
Cytopathic effect of PRRSV in MARC-145 cells post-treated with EGCG (**a**), EGCG palmitate (**b**), and ribavirin (**c**).

As shown in Figure 5, the cytopathic effect of PRRSV was reduced in MARC-145 cells post-treated with EGCG palmitate. The EC50 and SI values of EGCG, EGCG palmitate, and ribavirin are shown in Table 3. Greater inhibition of PRRSV was achieved with EGCG palmitate than with either EGCG or ribavirin, in accordance with their SIs.

**Table 3:** Cytopathic effect of PRRSV in MARC-145 cells post-treated with the test and control compounds.

Compounds	10 TCID_50_		100 TCID_50_	
	EC_50_ (μM) ^a^	SI ^b^	EC_50_ (μM)	SI
EGCG	9.18	257.01	97.88	24.11
EGCG palmitate	0.68	635.93	9.43	45.75
Ribavirin	0.44	212.71	3.95	23.82

### 2.6. Cytopathic Effect of PRRSV on MARC-145 Cells Co-Treated with the Test and Control Compounds

**Figure 6:**
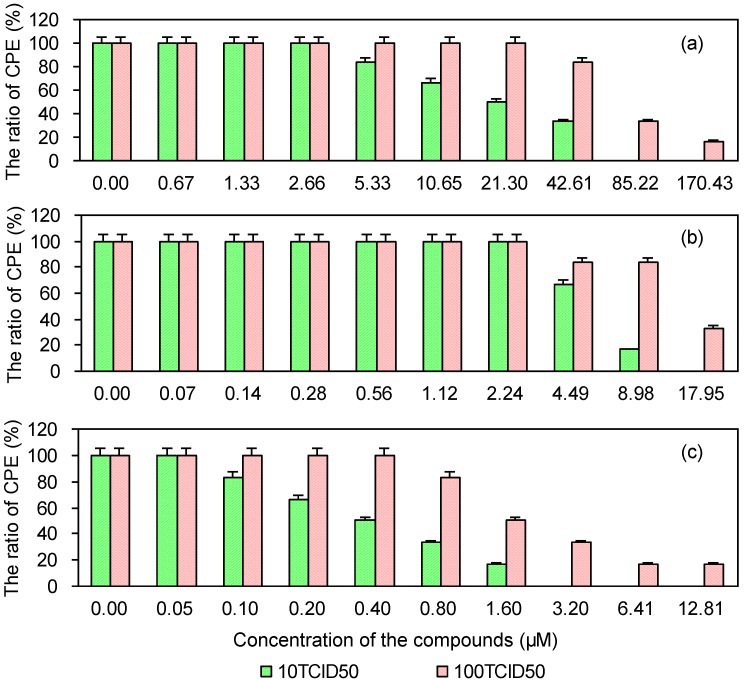
The cytopathic effect of PRRSV in MARC145 cells co-treated with EGCG (**a**), EGCG palmitate (**b**), and ribavirin (**c**).

As shown in Figure 6, in co-treated cells, EGCG palmitate inhibited PRRSV in a dose-dependent manner. The EC_50_ and SI values of EGCG, EGCG palmitate, and ribavirin in their inhibition of PRRSV are shown in Table 4. Ribavirin more strongly inhibited PRRSV activity than either EGCG or EGCG palmitate, in accordance with the SIs of the three drugs.

**Table 4:** Cytopathic effect of PRRSV in MARC-145 cells co-treated with the test and control compounds.

Compounds	10TCID_50_		100TCID_50_	
	EC_50_ (μM) ^a^	SI ^b^	EC_50_ (μM)	SI
EGCG	18.36	128.50	66.23	35.63
EGCG palmitate	5.86	73.64	12.69	33.99
Ribavirin	0.40	234.98	2.21	42.58

## 3. Discussion

EGCG exerts inhibitory effects on many viruses. At concentrations between 1 and 10 mM, EGCG inhibited the *in vitro* infectivity of both influenza A and influenza B virus in Madin-Darby canine kidney cells. An electron microscopy study showed that EGCG prevented viral adsorption to these cells [22]. Two recent studies found that EGCG inhibited the cellular attachment of HCV, thus disrupting the initial step of viral entry and suggesting both an antiviral strategy in the treatment of HCV infection and the prevention of HCV reinfection after liver transplantation [11,12]. EGCG also inhibited HIV-1 infectivity in human CD4(+) T cells by preventing the attachment of HIV-1 glycoprotein 120 to CD4 molecules on T cells [23]. However, EGCG is unstable in culture media, with a half-life of less than 30 min [15].

To increase the stability of EGCG, Mori *et al.* prepared a series of EGCG fatty acid monoesters and then demonstrated that, of these, the anti-influenza virus activity EGCG palmitate was dramatically enhanced. Specifically, the antiviral activity of EGCG palmitate against influenza A/PR8/34 (H1N1) virus was 24-fold higher than that of native EGCG [24]. Kaihatsu found that EGCG palmitate inhibited human and avian influenza A and B viruses, including those that were drug-resistant. EGCG palmitate was found to be more effective than neuraminidase inhibitors and was much better than zanamivir and osertamivir phosphate in inhibiting the infection of chicken eggs by avian influenza (H5N2) virus [25].

Based on previous *in vivo* and *in vitro* findings in the MARC-145 cell culture system, the CPE of aqueous extracts from teas on PRRS was assessed [26]. In that study, PRRSV was killed and the development of PRRS thus inhibited. In preliminary experiments, EGCG showed anti-PRRSV activity *in vitro*. Given the chemical instability of EGCG, in this study the more stable EGCG palmitate was synthesized and its anti-PRRSV activity was evaluated. In accordance with the SI values, our results showed that the anti-PRRSV activity of EGCG palmitate was the significantly greater than that of either EGCG or ribavirin. The anti-viral activity of EGCG palmitate was much stronger as a pre-treatment than as a co-treatment or post-treatment compound. Thus, EGCG palmitate may inhibit viral adsorption and cell intrusion.

## 4. Experimental

### 4.1. Materials and Chemicals

MARC-145 cells were obtained from BioHermes Bio-Pharmaceutical Technology Co., Ltd. (Wuxi, China). PRRSV was obtained from Dr. Yonggang Liu at the Harbin Veterinary Research Institute, Chinese Academy of Agricultural Sciences (Harbin, China).

EGCG (98%), ribavirin, dimethyl sulfoxide (DMSO), and 3-(4,5-dimethylthiazol-2-yl)-2,5-diphenyltetrazolium bromide (MTT), palm chloride, and pyridine were obtained from Sigma-Aldrich Shanghai Trading Co., Ltd. (Shanghai, China). Minimal essential medium (MEM) was purchased from Shanghai DoBio Biotech Co., Ltd. (Shanghai, China). Other chemicals were purchased from Beijing Chemical Reagents Company (Beijing, China).

### 4.2. The Synthesis, Preparation, and Identification of EGCG Palmitate

EGCG palmitate was prepared as described elsewhere [27]. Briefly, 0.92 g EGCG was added to 100 mL ethyl acetate, followed by 20 mL of palm chloride and 2.0 mL pyridine at room temperature. The ingredients were mixed in a magnetic blender for 5 h at 300 rpm.

After termination of the reaction, the sample was filtered through a 0.45-μm membrane and purified by silica gel column chromatography (silica gel and ethyl acetate/hexane as stationary and mobile phase, respectively). The absorbance of the eluent was measured at 280 nm in a UV spectrophotometer; according to the elution curve. The eluent corresponding to the main peak was combined, concentrated, and dried for identification. The predominant fraction was identified by MS (Esi, Absciex, Api-3000), IR (KBr, Nicolet, Magna-560), UV (Shimadzu, UV-2550), and ^1^H-NMR (CDCl_3_, Bruker, Avance-300 MHz). The isolated pure compound was used in the anti-PRRSV analysis.

### 4.3. Cell Culture

MARC-145 cells were cultured in a 25-cm^2^ cell culture flask in MEM containing 10% fetal bovine serum and 1% penicillin-streptomycin in a humidified incubator containing 5% CO_2_ at 37 °C. The cells were subcultured until they reached the exponential growth phase and then plated in 96-well culture plates for cytotoxicity and anti-PRRSV assays. The cells were propagated at 37 °C in an atmosphere of 5% CO_2_ [28].

### 4.4. Cytotoxicity Testing of EGCG, EGCG Palmitate, and Ribavirin

The cytotoxicity of EGCG, EGCG palmitate, and ribavirin was investigated using a 3-(4,5-dimethylthiazol-2-yl)-2,5-diphenyltetrazolium bromide (MTT) assay [29,30]. The cells were suspended in 200 μL of 2% MEM in 96-well plates. After 24 h of incubation, 200 μL of medium containing different concentrations of EGCG, EGCG palmitate, or ribavirin was added to the wells, followed by incubation for 72 h. The EGCG concentrations were 21.30, 42.60, 85.20, 170.40, 340.80, 681.59, 1,363.19, 2,726.38, and 5,452.75 μM. The EGCG palmitate concentrations were 4.49, 8.98, 17.95, 35.90, 71.80, 143.60, 287.20, 574.40, and 1,148.80 μM. The ribavirin concentrations were 1.60, 3.20, 6.40, 12.81, 25.62, 51.23, 102.46, 204.92, and 409.84 μM. Medium without any compound was used as a control. To evaluate cell viability, 20 μL of MTT solution (5mg/mL in NaCl) was added to the wells, which were further incubated for 4 h at 37 °C in a humidified atmosphere containing 5% CO_2_. After the remaining medium was removed, 150 μL of DMSO was added to each well to solubilize the precipitate. The resulting absorbance, which is proportional to the number of viable cells, was measured at 490 nm using a microplate reader (Awareness, STAT FAX 2100).

### 4.5. PRRSV Titration

MARC-145 cells were seeded onto 96-well plates for 24 h before infection. The PRRSV supernatants were 10-fold serially diluted, from 10^−1^ to 10^−9^, and 200 μL of each dilution was added to each of six wells. Cell maintenance medium without PRRSV was used as a control. The cells were incubated at 37 °C for 72 h in an incubator containing 5% CO_2_. The cell cytopathic effect (CPE) was observed daily under an inverted microscope [31]. The 50% tissue culture infective dose (TCID_50_) was calculated by the Reed–Muench method [21]. 

### 4.6. The Effect of EGCG Palmitate on PRRSV *in Vitro*

The effect of EGCG palmitate on PRRSV was investigated in three different experiments.

**Pre-treatment with EGCG palmitate:** On the basis of the maximal non-cytotoxic concentration, EGCG palmitate was prepared and 200 μL was added to a monolayer of MARC-145 cells plated in 96-well plates. The plates were then incubated at 37 °C for 4 h in an atmosphere containing 5% CO_2_. The EGCG palmitate solution was removed and replaced with the virus solution, which was allowed to adsorb for 1.5 h. The virus solution was then removed and the cells were washed once with NaCl; subsequently, 200 μL of cell maintenance medium was added to each well and the plates were incubated for 72 h as described above. The CPE was then determined.

**Post-treatment with EGCG palmitate:** A monolayer of MARC-145 cells plated in 96-well plates was treated with 200 μL of EGCG palmitate and the plates were incubated at 37 °C for 1.5 h in an incubator containing 5% CO_2_. The virus solution was removed and the cells were washed once with NaCl. Subsequently, 200 μL of the different concentrations of EGCG palmitate were added to each well and the plates were then incubated at 37 °C for 72 h in an incubator containing 5% CO_2_. The CPE was then determined [31].

**Co-treatment with EGCG palmitate:** EGCG palmitate was mixed with isometric virus solution and the mixture was incubated at 37 °C for 2 h. A monolayer of MARC-145 cells plated in 96-well plates was then treated with 200 μL of the mixture and the plates were incubated as described above for 1.5 h. The supernatant was removed and the cells were washed once with NaCl. Subsequently, 200 μL of cell maintenance medium was added to each well and the plates were incubated for 72 h in 5% CO_2_ at 37 °C. The CPE was then determined.

Each process in the three above-described experiments was repeated six times. The controls consisted of cells treated in the same way but with EGCG or ribavirin. The antiviral effect is expressed as the concentration that decreased the virus-induced CPE by 50% (50% effective concentration, EC_50_).

## 5. Conclusions

In this work, EGCG palmitate was shown to possess dose-dependent anti-PRRSV activity *in vitro*. The cytotoxicity of EGCG palmitate was higher than that of EGCG but its anti-PRRSV activity was significantly higher than that of either EGCG or ribavirin when added as a pre- or post-treatment. The results were in agreement with the determined SI values. In particular, the anti-PRRSV activity of EGCG palmitate was much stronger when the drug was added as a pre-treatment than as either a post- or co-treatment. Thus, EGCG palmitate is of interest to the pharmaceutical industry since administration of this inhibitor may prevent PRRSV infection in pigs.
